# Utilizing Ras Signaling Pathway to Direct Selective Replication of Herpes Simplex Virus-1

**DOI:** 10.1371/journal.pone.0006514

**Published:** 2009-08-04

**Authors:** Weihong Pan, Vidya Bodempudi, Tuba Esfandyari, Faris Farassati

**Affiliations:** 1 The University of Minnesota Medical School, Minneapolis, Minnesota, United States of America; 2 Department of Medicine, The University of Kansas Medical Centre, Kansas City, Kansas, United States of America; Emory University, United States of America

## Abstract

Re-engineering the tropism of viruses is an attractive translational strategy for targeting cancer cells. The Ras signal transduction pathway is a central hub for a variety of pro-oncogenic events with a fundamental role in normal and neoplastic physiology. In this work we were interested in linking Ras activation to HSV-1 replication in a direct manner in order to generate a novel oncolytic herpes virus which can target cancer cells. To establish such link, we developed a mutant HSV-1 in which the expression of ICP4 (infected cell protein-4, a viral protein necessary for replication) is controlled by activation of ELK, a transcription factor down-stream of the Ras pathway and mainly activated by ERK (extracellular signal-regulated kinase, an important Ras effector pathway). This mutant HSV-1 was named as Signal-Smart 1 (SS1). A series of prostate cells were infected with the SS1 virus. Cells with elevated levels of ELK activation were preferentially infected by the SS1 virus, as demonstrated by increased levels of viral progeny, herpetic glycoprotein C and overall SS1 viral protein production. Upon exposure to SS1, the proliferation, invasiveness and colony formation capabilities of prostate cancer cells with increased ELK activation were significantly decreased (p<0.05), while the rate of apoptosis/necrosis in these cells was increased. Additionally, high Ras signaling cells infected with SS1 showed a prominent arrest in the G1 phase of the cell cycle as compared to cells exposed to parental HSV-1. The results of this study reveal the potential for re-modeling the host-herpes interaction to specifically interfere with the life of cancer cells with increased Ras signaling. SS1 also serves as a “prototype” for development of a family of signal-smart viruses which can target cancer cells on the basis of their signaling portfolio.

## Introduction

Ras is a major proto-oncogene involved in 35% of all human cancers (Adjei, 2001). Ras activation results in stimulation of different mitogen-activated protein kinases (MAPKs) [1], [2], [3], [4]. MAPKs are involved in diverse cellular functions including cell proliferation, cell cycle regulation, cell survival, angiogenesis, and cell migration [1], [5], [6], [7], [8], [9], [10], [11], [12]. Different extracellular chemical and physical signals can stimulate MAPKs making them an important part of the machinery needed to transduce signals from receptor to regulatory molecule inside the cell. Activation of the Ras/Raf/ERK1/2 pathway results in the serine/threonine kinase ERK1/2 phosphorylating, among other substrates, the nuclear transcription factor, ELK [13], [14]. ELK is a member of the Ets-family and is a component of the ternary complex that mediates gene activity in response to serum and growth factors. Phosphorylated-ELK, in combination with serum response factor (SRF), binds to an enhancer element in the c-fos promoter referred to as serum response element (SRE) inducing the transcription of many genes involved in biological functions such as proliferation and differentiation [15], [16].

We have shown previously that cells with overactivation of Ras signaling are more permissive to infection by herpes simplex virus-1 (HSV-1) due to the impaired action of double-stranded RNA-induced protein kinase (PKR), the main host defense mechanism against viral infection [17], [18]. Here, we expand on these studies by establishing a direct relationship between Ras signaling in host cells and HSV-1 replication by developing a mutant HSV-1 which is responsive at transcriptional level to Ras activation. Our goal was to engineer the pathogenicity of the virus to only interfere with host viability on the basis of overactivation of the Ras/ERK/ELK pathway. This mutant virus is referred to as Signal-Smart 1 (SS1) virus and was tested in a range of prostate cancer cells. Prostate cancer has been tested before as a target for gene therapy using an amplicon herpes system (containing a probasin-derived promoter) to complement a replication defective mutant herpes[19]. In this study we show that the SS1 virus preferentially infects prostate cancer cells with increased ELK/SRE activation inducing changes in viability, invasiveness, colony formation, cell cycle progression, apoptosis and necrosis.

## Results and Discussion

### Construction of the mutant SS1 virus

**Figure 1:**
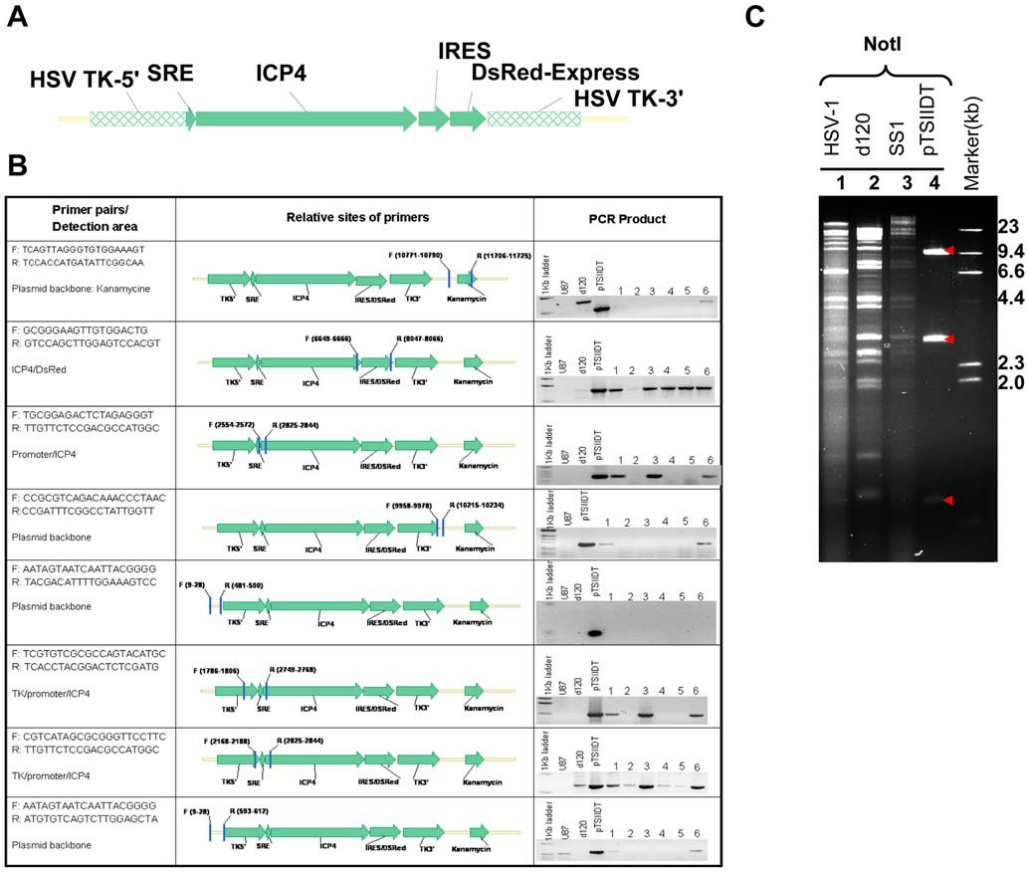
Characteristics of Signal-Smart 1(SS1) mutant HSV-1. The SS1 virus contains one copy of ICP4 gene under the control of 5xSRE and minimal TATA sequence. (A) Viral genomic PCR reactions confirm the structure of the recombinant ICP4 gene. The sequence for each pair of primer, the relative positions and of the primers as well as their sequence and results are shown. The PCRs were performed on U87 cells (non-transfected), d120-genomic DNA (labeled as d120) and the plasmid pTSIIDT. Performing PCR on back-bone elements of pTSIIDT is done in order to rule out the contamination of genomic viral DNA preparations by pTSIIDT. (B) Genomic structure of HSV-1 and SS1 virus and pTSIIDT plasmid DNA. NotI restriction analysis: The 9.3 Kb NotI fragment contains the plasmid backbone, the 2.8 and 0.7 Kb fragments are the expected digestion pattern from both SS1 and pTSIIDT, which contains the ICP4 gene (arrowheads). Twenty µg DNA of each sample was digested over night at 37°C and resolved in 1% agarose gel. Lane 1:HSV-1/NotI; Lane 2:d120/NotI; Lane 3:SS1/NotI; Lane4: pTSIIDT/NotI; Lane5:DNA marker (from top to bottom: 23, 9.4, 6.6, 4.4, 2.3, 2.0 and 0.5 Kb).

The SS1 mutant virus contains one copy of the herpetic alpha-4 gene under the control of a synthetic promoter. This gene encodes the infected cell protein-4 (ICP4), which is a necessary protein for viral replication. The synthetic promoter is composed of five tandem repeats of SRE (the DNA binding element for ELK) and a minimal TATA sequence. The 5xSRE-TATA-ICP4 sequence is followed by an internal ribosome entry site (IRES) and the fluorescent protein DsRed-express (Figure 1A). The SS1 virus was produced by infecting E5 cells (expressing ICP4) with d120, a mutant HSV-1 with deletions in both copies of the alpha-4 gene, followed by transfection of d120-infected E5 cells with a plasmid containing 5xSRE-ICP4-IRES-DsRed-express (pTSIIDT). The 5xSRE-ICP4-IRES-DsRed-express construct is flanked by two complementary regions homologus to HSV-1 thymidine kinase (TK). Once both d120 viral genomic DNA and pTSIIDT are present within the E5 cells, homologus recombination with the TK gene results in the insertion of 5xSRE-ICP4-IRES-DsRed-express in the d120 genome and generation of the SS1 virus.

Figure 1B explains the results of PCR experiments on SS1 DNA preparations. Six independent viral isolates (numbered 1–6) are investigated with the purpose of confirming the structure of this mutant virus. The structure of the recombinant promoter was confirmed by performing PCR using primers which span over the promoter (5xSRE-TATA) and cover a portion of the ICP4 gene. In each case, the PCR product was sequenced and compared to the related sequence in our database. Contamination of genomic viral DNA preparations by pTSIIDT was ruled out by performing PCR targeting the backbone areas of this plasmid. Following analysis of viral structure by these experiments, isolate number 3 was selected for further expansion to be used in the experiments. Furthermore, the genomic DNA from d120, SS1 and HSV-1 were analyzed by restriction digestion with NotI (Figure 1C, results shown for NotI). The segment analysis by these restriction endonucleases confirmed that the inserted gene sequences in SS1 were structurally consistent with the plasmid, pTSIIDT and that overall genomic structure of SS1 remains in harmony with d120 and wild-type HSV-1. The SS1 virus was then amplified and titrated with known methods [20].

### Prostate cells contain different levels of Ras/ERK/ELK activation

**Figure 2:**
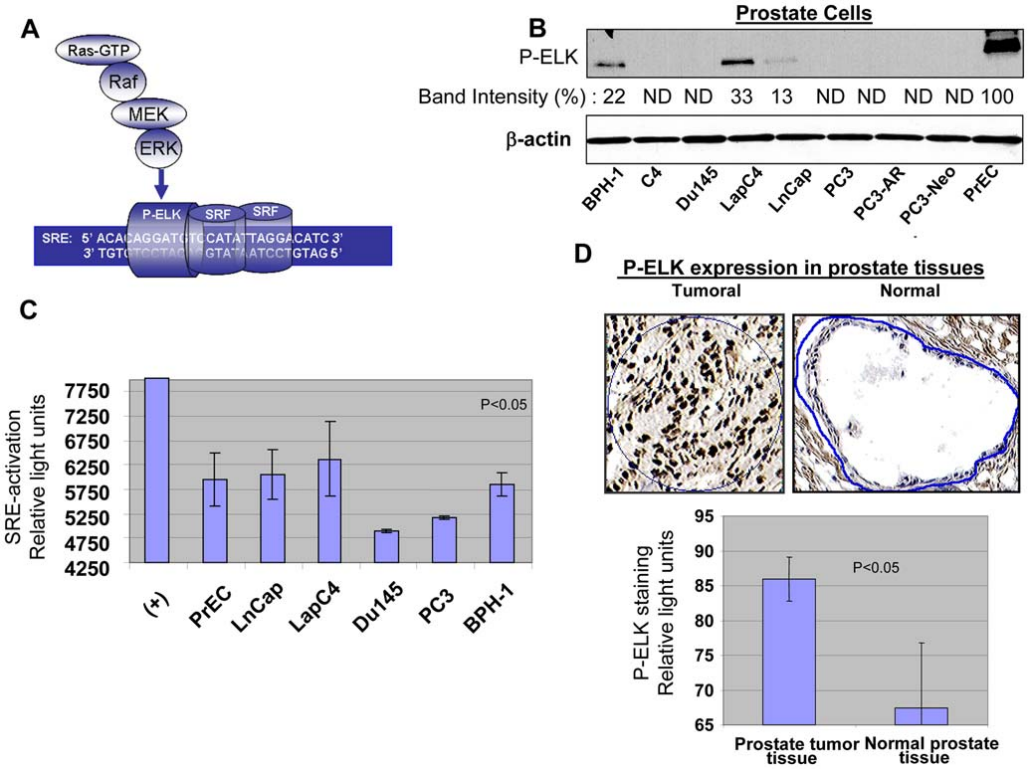
Ras signaling and activation of ERK/ELK/SRE in prostate cells and tissue. Activation of Ras leads to initiation of a signal via Raf/MEK/ERK which in turn phosphorylates/activates transcription factor ELK. ELK in combination with SRF drives expression from promoters containing SRE elements. (A) Activation of ERK and phosphorylation of ELK in a series of prostate cells including prostate cancer cells, PrECs and BPH-1 is evaluated by in-vitro kinase assay for P-ERK. Band intensities are evaluated in comparison to the strongest band which in this case was the band for PrEC represented as 100%, ND stands for not detectable. (B) Activation of SRE by binding of P-ELK was evaluated by performing a Luminex-based assay. Existence of P-ELK and its binding to SRE elements protects these oligomers from digestion by a nuclease and allows generation of light. (C) Expression of P-ELK (active ELK) in malignant prostate tissue samples (n = 10, average Gleason score 6.0) and non-malignant prostate tissue was evaluated by performing immunohistochemistry and following image analysis for dark brown nuclear staining representing P-ELK using appropriate software. Upper panels show a sample of P-ELK staining for tumoral and normal tissues while the lower panels show average of staining intensity for P-ELK in different samples.

We next evaluated the level of Ras/ELK signaling in malignant and non-malignant prostate cells. Phosphorylated ELK and activation of SRE are both down-stream effectors of the Ras/ERK/ELK pathway (Figure 2A). Levels of both effectors were measured in several prostate cancer cell lines (PC3, C4, C4-2, Du145, LapC4, LnCap, PC3-AR, PC3-Neo), primary prostate epithelial cells (PrEC), benign prostate hyperplasic cells (BPH-1), and human tissue samples. A higher level of phospho-ELK (P-ELK) was seen for the prostate cancer cell lines LapC4 and LnCap and the non-malignant cell lines BPH-1 and PrEC (Figure 2B, band intensities are evaluated in comparison to the strongest band which in this case was the band for PrEC represented as 100%, ND stands for not detectable). For the rest of this study, we focused on LapC4 and LnCap as high Ras signaling cancer cells, Du145 and PC3 as low Ras signaling cancer cells, along with PrEC and BPH-1 cells. We next measured the binding of phospho-ELK to SRE (as a representation of SRE activation) in these cells using a Luminex based assay (Figure 2C). LnCap and LapC4 exhibited higher levels of SRE activation than Du145 and PC3 cells. PrEC and BPH-1 also showed increased SRE activation. It is important to note that while Figure 2B evaluates the levels of P-ELK generated by ERK-pathway, figure 2C provides us with activation levels of SRE in these cells. Although ERK is the major contributor to ELK phosphorylation, other pathways might also induce ELK and therefore, at least partly, contribute to SRE activation shown in figure 2C. In any case, LnCap, LapC4, BPH-1 and PrEC on both assays exhibit higher levels of activation than Du145 and PC3.

Given that PrECs showed higher levels of phospho-ELK than any of the prostate cancer cell lines, we wanted to determine if this finding was in harmony with phospho-ELK levels in tumoral and normal prostate tissues. To achieve this, the levels of phospho-ELK activation in prostate tissues were compared in tumoral (n = 10, average Gleason score over 6.0) and non-tumoral (n = 10) samples with immunohistochemistry using an antibody specific for phospho-ELK. Dark brown staining in nucleus represents phospho-ELK and was quantified by scanning slides using related software. Levels of phospho-ELK were significantly lower in normal tissue samples as compared to malignant samples (Figure 2D) (p<0.05). Therefore, although PrECs show elevated levels of ELK activation, normal prostate tissue remains significantly lower in this regards as compared to tumoral tissue. These results confirm that a major difference exists between information obtained from cultured cells *in- vitro* and analysis of tumoral tissues.

### Prostate cells with elevated levels of ELK/SRE activation are more permissive to SS1 virus

**Figure 3:**
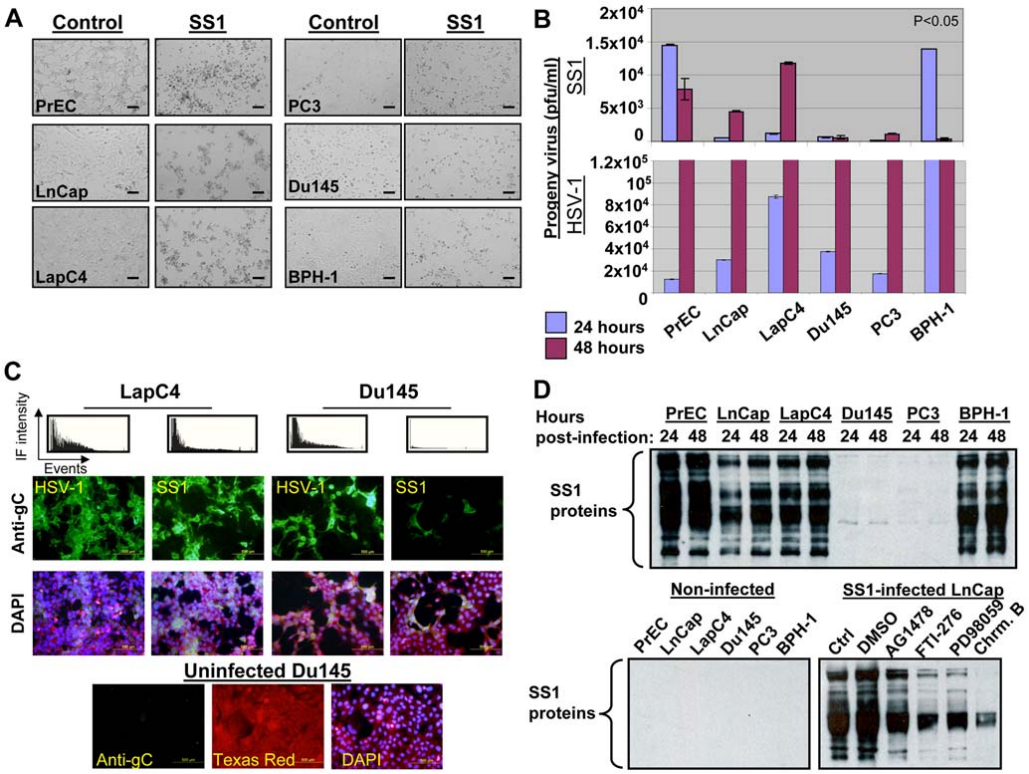
SS1 virus preferentially infects cells with increased Ras/ELK signaling. Morphological studies of SS1-infected cells (MOI∼1) show rounding and clumping (signs of herpetic infection) in cancer cells with elevated Ras/ELK signaling (LnCap and LapC4) as well as PrEC and BPH-1 cells as compared with Du145 and PC3 cells at 24-48 hours post-infection (MOI∼1). (A) The titration of viral progeny at 24–48 hours post-infection (MOI∼1) revealed enhanced levels for LnCap and LapC4 as well as PrEC and BPH-1 as compared with Du145 and PC3 cells. In case of PrEC and BPH-1 cells titrations maximize at 24 hours. (B) Immunofluorescent (IF) studies for glycoprotein C (gC, a marker for herpetic infection) revealed enhanced expression for LapC4 (high Ras) as compared to Du145 (low Ras) cells upon infection with SS1 (second row). Expression of this marker in these two cell lines was at much closer levels once these cells were infected with parental HSV-1. Top panel represents IF intensity for each panel versus number of captured events in each field. Third row represents the DAPI staining of related panels. Bottom panel shows staining of uninfected Du145 cells and the background Texas-red and DAPI staining. (C) The expression of SS1 proteins was investigated by western blotting on lysates from infected and control cells using an anti-body raised against all HSV-1 antigens. Higher and more comprehensive levels of SS1 protein synthesis was observed for LnCap, LapC4, PrEC and BPH-1 as compared to Du145 and PC3 cells. Controls (uninfected cells) show no bands proving the specificity of antibody for viral proteins. Lower panel shows a significant decrease in the expression of SS1 proteins in infected LnCap cells upon exposure to inhibitors of Ras (FTI-277 at 20 µM), MEK (PD98059 at 25 µM) and ELK (Chromomycin A at 10 µM) but not the vehicle (DMSO). A lower level of inhibition was observed for inhibitor of EGFR, AG1478 (0.5 µM). Cells were incubated with these inhibitors overnight and then exposed to SS1 while inhibitors existed in the media.

In order to understand the effect of high and low Ras signaling on permissiveness, we infected prostate cancer cells and non-malignant PrECs and BPH-1 cells with SS1 and parental HSV-1 virus at a multiplicity of infection of 1 (MOI∼1). A variety of techniques including titration of viral progeny, immmunoflourescent (IF) studies on envelope glycoprotein C (gC, an standard marker for herpetic infection) and western blotting for herpes proteins were used to evaluate and compare the permissiveness of these cells. Morphological studies of SS1-infected cells showed a more prominent pattern of infection (rounding and clumping) for LnCap, LapC4, PrEC and BPH-1 as compared with Du145 and PC3 cells (Figure 3A). Levels of SS1 progeny virus detected upon infection of LapC4 and LnCap (high Ras cancer cells) were higher than Du145 and PC3 (low Ras cancer cells) (Figure 3B). PrECs and BPH-1 also produced elevated levels of viral progeny because of their elevated ELK activation as mentioned before. In case of HSV-1, however, progeny virus levels were found to be comparable in all cells at 48 hours post-infection (Figure 3B lower panel). Another level of evidence for the enhanced capability of SS1 to target cells with increased Ras/ELK signaling was obtained by studying these cells for expression levels of gC. Although HSV-1 infected LapC4 and Du145 with comparable intensity, LapC4 cells were remarkably more permissive to the SS1 virus in comparison to Du145 (Figure 3C). Lower panel shows staining of uninfected LapC4 cells with anti-gC antibody in order to prove its specificity for infected cells (Texas red is the background stain of the anti-gC antibody preparation and serves to visualize the cell monolayer).

Finally, evaluating expression of a series of herpetic proteins in these cells by western blotting for viral proteins (Figure 3D) revealed enhanced and comprehensive protein expression in PrECs, BPH1, LapC4 and LnCap as compared to minimal levels of viral protein synthesis for Du145 and PC3 cells (all bands show herpetic proteins). If replication of SS1 is dependent on Ras signaling, it would be rational to expect that inhibitors of this pathway would reduce the production of herpetic proteins and therefore permissiveness to the virus. Indeed, once exposed to the inhibitors of Ras signaling a reduction in the levels of SS1 protein synthesis was observed for LnCap cells. The reducing effects of EGFR inhibitor AG1478 (0.5 µg) was found to be lesser than effects observed for FTI-276(blocker of Ras farnesylation used at 5 nm), PD98059 (blocker of MEK/ERK pathway used at 20 µM) and Chromomycin A (a blocker of ELK binding to SRE used at 500 nM). Interestingly, the effects of Chromomycin A [21], was found to be the strongest providing further evidence for dependency of SS1 virus on transcriptional activity of ELK. All of these data show the capability of the SS1 virus to preferentially target cells with elevated Ras/ELK signaling. To this end, it is important to note that PrEC cells, although not transformed but containing enhanced levels of ELK/SRE activation, were not found to be immune to the effects of the virus. We attribute such a phenomenon to the altered signaling characteristics of primary cells once cultured *in vitro*. This claim is supported by the significantly lower levels of ELK activation in normal prostate tissue as compared to tumoral samples (Figure 1D).

### Prostate cells with elevated levels of ELK/SRE activation were less viable and invasive upon exposure to SS1 virus

**Figure 4:**
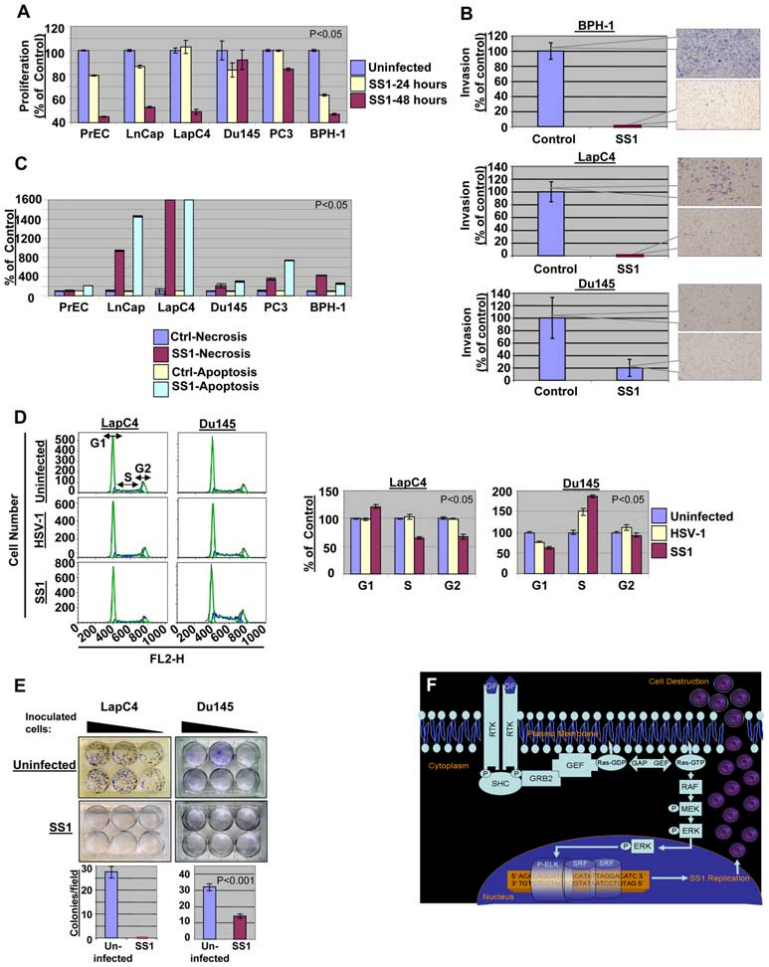
The effects of SS1 infection on the phenotype of prostate cells. The proliferation rate of prostate cells is decreased upon exposure to SS1 virus (MOI∼1) with lower levels observed in case of prostate cancer cells with high Ras signaling (LnCap and LapC4) and PrEC and BPH-1 cells as compared to Du145 and PC3 cells (low Ras cells). (A) Invasiveness of prostate cancer cells LapC4 and Du145 as well as BPH-1 cells were decreased after 24 hours of exposure to SS1 virus in a significant manner. The decrease in invasiveness was more prominent for cells with elevated Ras signaling (LapC4 and BPH-1) as compared with Du145. Control Du145 cells were much less invasive than other cells. (B) Increase in necrosis and apoptosis is observed upon exposure of prostate cells to SS1 virus. The induction in necrosis/apoptosis is remarkably greater in LnCap and LapC4 cells. (C) The progression of cell cycle is altered upon exposure of prostate cancer cells to SS1 virus. In case of a high Ras cell (LapC4) an increase in G1 and decrease in S and G2 was observed upon infection with SS1 as compared with parental virus infected and control cells. Du145 (a low Ras cell), however, showed a passage through G1 but significant enhancement of S1 fraction. Left panels represent captured data plotted as fluorescence intensity (FL2-H channel) versus cell number for different phases of cell cycle. The right panel portrays these data as percentage of control (non-infected cells) for SS1 or HSV-1 infected cells. (D) Colony formation capability of LapC4 and Du145 cells was also significantly reduced upon infection with SS1. Upper panels show formation of colonies for a range of number of inoculated infected cells. The lower panels show average number of colonies per microscopy field for SS1-infected and control groups. The colony forming capability of Du145 cells were less inhibited as compared to LapC4 cells. (E) The mechanism of SS1 action is illustrated in this figure. Activation of Ras signaling pathway stimulates a signal through Raf/MEK/ERK pathway inducing phosphorylation of ELK. Stimulation of SRE elements by a complex including P-ELK results in expression of ICP4 and replication of SS1 which eventually destroys the cell via infection.

In next step we studied the biological outcome of exposing prostate cells to SS1 virus (MOI∼1). The proliferation rate of prostate cells was measured at 24 and 48 hours post-infection and compared to uninfected controls. As in figure 4A, once exposed to the SS1 virus, the proliferation rate of cells with elevated Ras/ELK signaling is more prominently reduced as compared with cancer cells with lower Ras/ELK signaling (p<0.05).

The effects of SS1 infection on invasiveness of prostate cancer cells was also studied by testing the capabilities of BPH-1, LapC4 and Du145 in passing through a layer of Matrigel *in vitro* in comparison to uninfected controls at 24 hours after infection. Figure 4B shows that a complete blockade of invasiveness was observed for SS1 infected BPH-1 and LapC4 (top and middle panels). The invasiveness of Du145 cells was also reduced; however, even cells in the control group were not capable of invading through matrigel with a comparable intensity as the other tested cells (bottom panel). Therefore, the SS1 virus is capable of specifically targeting prostate cells with increased Ras signaling and thereby specifically inducing loss of viability and invasiveness in these cells. Both of these biological effects on the host cell can play an important role in the translational potentials of SS1 for targeting cancer cells.

### Infection with SS1 enhances cell death, alters cell cycle progression and reduces colony formation of prostate cancer cells

In order to investigate the mechanism of cell death upon exposure to SS1, we evaluated the occurrence of necrosis and apoptosis in these cells once exposed to SS1 virus. Figure 4C shows a significant (p<0.05) increase in both necrosis and apoptosis upon infection of LnCap and LapC4. Although all other cells showed apoptosis and necrosis, the increase observed in the case of cancer cells with elevated Ras signaling (LapC4 and LnCap) was remarkably greater than other cells. Interestingly, PrECs seem to mainly undergo apoptosis upon infection with SS1.

In next step we evaluated the outcome of exposure to SS1 virus on cell cycle progression of high and low Ras signaling cells and compared these results with the effects of parental HSV-1 infection. Figure 4D shows the results of cell cycle analysis after 24 hours of exposure to MOI∼1 of SS1 or HSV-1 virus as well as data obtained from uninfected controls. In LapC4 cells SS1 infection seemed to cause an increase in the population of cells in the G1-phase of the cell cycle (p<0.05). HSV-1, however, did not induce such effects in these cells. In the case of Du145 cells, a reverse phenomenon was observed as the fraction of G1 cells was decreased, while cells in S-phase increased.

Although the functional analysis of these data requires further evaluation, it is possible to interpret them in a preliminary manner in light of our understanding of the role of Ras signaling in the passage of cells from G0 to S-phase and the impact of herpetic infection on this passage. Ras signaling elevates levels of cyclin-D1, which is responsible for facilitating advancement through G1-phase. Ras signaling influences the transcription, translation and protein stability of cyclin D1. An increase in p21 levels results from direct effects on transcription and indirect effects through cyclin D1 on protein stability. Cyclin D1 and p21 then drive the formation of active cyclin-D1–CDK4 (cyclin-dependent kinase-4) and cyclin-D1–CDK6 complexes, which promote cell-cycle progression by phosphorylating retinoblastoma (RB) proteins [22]. The Ras-Raf-ERK pathway has been shown to be required for expression of cyclin D1 and its assembly into a complex with CDK4 or CDK6. On the other hand, HSV-1 infection has been shown to prevent infected cells from progression from G1 into S-phase diminishing the normal increase in Cyclin D1 and D3 [23], [24]. SS1 virus, with its replication dependency on Ras signaling, will be more restrictive to G1/S transition since it produces ICP4 (a protein shown to be required for viral-induced G1/S blockade) under direct influence from Ras/ERK/ELK pathway. In the case of such low Ras signaling cells as Du145, the SS1 virus is less effective in blocking G1/S transition resulting in an increase in the S fraction. Therefore, the passage through G1/S in Du145 cells is more efficient in SS1-infected cells as compared to HSV-1-infected cells due to the lesser infectibility of Du145 cells by the SS1 virus.

Finally, the capabilities of LapC4 and Du145 cells to form colonies upon infection with SS1 (MOI∼0.5) virus was evaluated. Figure 4E shows complete abrogation of the capabilities of LapC4 cell lines after three weeks of inoculation of cells for this assay. In case of Du145 cells, a decrease was observed in the number of colonies upon exposure to SS1 however a complete blockade was not observed. Reduction in the colony formation capabilities of prostate cancer cells is another indicator of change in the malignant phenotype of prostate cancer cells upon exposure to the SS1 virus.

### Concluding Remarks

Figure 4F portrays the overall Ras-dependent mechanism for SS1 replication. Ras signaling can be activated by a variety of up-stream events such as binding of growth factors to the receptor tyrosine kinases (RTKs) or pro-oncogenic activation of Ras or signaling via down-stream molecules such as Raf or ERK. Activation of Ras signaling then results in activation of ELK. As a consequence of elevated levels of Ras signaling, therefore, the SS1 virus replicates efficiently due to the existence of ELK binding sites in the promoter region of its recombinant alpha-4 gene. Correlation between Ras signaling and SS1 replication and induction of cytotoxic effects such as loss of viability and induction of necrosis and apoptosis was shown in our work. Additionally, invasiveness was seen to be blocked as early as 24 hours post-infection with SS1. While PrECs used in our studies also showed elevated levels of ELK activation, their signaling may not reflect the situation in tumor tissues based on the data shown in our work and others [25], [26]. Also, once exposed to SS1, high Ras prostate cancer cells had an increase in the G1 fraction of cells and lost their colony formation capabilities. Although the interaction between two complex biological systems (virus and host) is a function of multi-factorial events, the linkage of expression of ICP4 to the Ras pathway seems to direct the overall dynamics of this relationship, which is significantly influenced by Ras signaling. Further evaluation of the effects of signal-smart viruses on host cell biology improves our understanding about the mechanism of the virus-host relationship and opens new horizons in using these biological agents in detection and targeting cancer cells with enhanced specificity and efficiency.

## Materials and Methods

### Cells, media, chemicals and viruses

African green monkey kidney cells (Vero) and E5 (Vero cells transfected with HSV-1 alpha-4 gene) cells were grown in Dulbecco's modified Eagle's medium (DMEM) supplemented with 10% Fetal calf serum. The prostate cancer cell lines were grown in BPH-1 (RPMI 1640+5%FBS+1%A.B), Du145 (DMEM+10%FBS+1% AB), LapC4 (IMDM+10%FBS+1% AB), LnCap (RPMI-1640+10%FBS+1% AB) and PC3 (Hams F-12K+10%FBS+1%AB). HSV-1(F) virus was a gift from Dr. Bernard Roizman (University of Chicago). HSV-1 mutant d120, containing a 4.1-kb deletion in both copies of the ICP4 gene, and the complementary cell line E5 [27], [28] were kindly provided by Robert L. Martuza (Harvard Medical School, Boston, MA). The stock of mutant d120 virus was prepared in E5 cells. Every stock of d120 virus was titrated by infecting 10^6^ PFU of viruses from the stock in Vero monolayer, harvesting the culture medium, and then determining the existence of any infectious progeny by plaque assay on Vero cells.

### Plasmid construction

Using a sense primer, 5′-CTCACAGCTAGCTTGGGTAACGCCAGGGTTTTC-3′ and antisense primer, 5-′ACTGTATAGATCTCGCCCCTCGAATAAACAACGC-3′, we amplified the ICP4 gene by PCR from DNA template, pGH108. The PCR product (4.2 KB) was then inserted into the vector, pCR-Blunt-TOPO (Invitrogen, catalog no:K2800-20). An EcoRI-ICP4-EcoRI fragment from the TOPO clone was then isolated and subcloned into the vector, pIRES/DsRed express (Clontech, catalog no:632463), between CMV and IRES/DsRED genes at EcoRI single site. The construct was designated as pIID. In next step, we amplified part of human TK gene from DNA template, pHSV106, by using primers, 5′-ATCGCTAGCTCCAAGACTGACACATT-3′ and 5′-ATGCTAGCACTAGTACCGGTAGTACTGCTGAGGTGGGCTTTGGACGTCTT-3′. The PCR product was then cloned into the vector, pCRII-TOPO (Invitrogen, Cat. No. K4600-01). A NheI-TK-NheI fragment, TK5′, from the TOPO-clone was then subcloned into the plasmid, pIID, in front of ICP4 gene. The construct was designated as pTIID. The 3′ segment of the viral TK gene was also amplified from pHSV106 by primers 5′-GGGTTAACATTTAAATCAGGTCGCCGTTGGGGGCCA-3′ & 5′-GGGTTAACAAATGAGTCTTCGGACCTC-3′ and subcloned into the pCRII-TOPO as mentioned above. The HpaI-TK-HpaI fragment, TK3′, was cloned into the plasmid, pTIID, on the 3′of the IRES/DsREd genes at its HpaI single site. This construct was designated as pTIIDT. As mentioned above, we did TOPO-Clone for the SRE promoter from the template DNA, pSRE-Luc, by primers, 5′-CCTCAGCTGTCTGGATCCAAGCTAGGA-3′ and 5′-GACTAGTATGCCAAGCTGGAA TTCGAG-3′. A BbvCI-SRE-SpeI fragment was cloned into the plasmid, pTIIDT, between TK 5′ and ICP4 gene. The construct was designated pTSIIDT and was eventually used for transfecting E5 cells. All the fragments from PCR TOPO clones were sequenced.

### Generation of SS1

E5 cells were seeded onto 150 mm dish to grow overnight to 80%–90% confluency.

Transfection of pTSIID was performed by using LipofectamineTM2000 (Invitrogen, CA) according to the manufacturer's protocol. After transfected E5 cells were incubated at 37°C and 5% CO_2_ for 72 h, the supernatant was discarded and transfected cells were infected with d120 virus by adding 10 ml diluted d120 preparation for 1.5 h infection incubation (with gentle rocking). Fresh medium was then added and incubation continued for three days. The virus was then harvested by scraping cells off the flask surface in combination with supernatant followed by three cycles of freeze-thaw and supplementation with 10% autoclaved non-fat milk for increased stability. Recombinant viral isolates were purified by three rounds of plaque purification on Vero cells. Total and viral genomic DNA was isolated for each recombinant virus isolate (six independent viral isolates numbered 1–6 are shown in figure 1B). Using PCR reactions by different primer pairs, the genomic composition of these recombinant virus isolated were identified with established methods [20]. Isolate number 3 was chosen for expansion and completion of experiments in this manuscript.

### Nuclear extraction and SRE activation Luminex assay

Nuclear extract was made by a nuclear extraction kit from Marligen Bioscience (cat. #11906-100). Briefly, cells were cultured in a T-175 flask to about 90% confluency and washed twice with ice-cold PBS, collected with a cell scraper in PBS and transferred to a 15 ml conical tube. The cells were then centrifuged 5 minutes at 3000 rpm at 4°C and pellet was resuspended in 500 µl complete hypotonic lysis buffer and incubated on ice for 10 minutes. In next step 25 µl of detergent solution was added, vortexed for 5 seconds and spun for 5 minutes at 3000 rpm at 4°C. The supernatant was then discarded and pellet was washed twice with 500 µl complete wash solution. 50 µl of complete Extraction Buffer1 and 50 ul of complete extraction buffer 2 were then added and the mixture was then incubated on ice for 30 minutes. The nuclear extract was clarified by centrifugation and stored at −80°C until use. Three µg nuclear extract was used for SRE transcription assay by a kit from Marligen Bioscience (catalog # 11944-096 and 11906-100) according to manufacturer's instructions. Briefly nuclear extracts were incubated with a mixture of biotinylated DNA binding probes. During the incubation, the active transcription factor complexes in the sample were bound to their specific probes, whereas unbound probes were removed utilizing Marligen's patented digestion step. The remaining probes were incubated with a mixture of different fluorescently dyed xMAP® beads. Each different xMAP® bead is coupled with a unique probe that recognizes a specific DNA binding probe so that the probes hybridize to their specific bead region. Following hybridization, the samples were incubated with streptavidin-phycoerythrin (SAPE) and read on the Luminex™ instrument, which detect the specific transcription factors activated in the sample by their unique bead region and quantified by the intensity of the SAPE signal.

### SDS-PAGE and Western blot analysis

Different cells were lysed with a single detergent lysis buffer [50 mM Tris (pH 8.0), 150 mM NaCl, 0.02% sodium azide, 100 µg/ml phenylmethy-sulfonyl fluoride, 1 µg/ml aprotinin, and 1% Triton X-100], normalized for the amount of total protein and subjected to SDS-PAGE using BioRad mini-cell protein-II system (using pre-cast 10% discontinuous gels) followed by electroblotting onto nitrocellulose paper. The membrane was then washed and incubated with a primary rabbit antibody against all HSV-1 antigens (Dako, CA), followed by the horseradish peroxidase (HRP)-conjugated secondary antibody. After extensive washing, the blot was exposed to Lumigel detection solution and subjected to autoradiography.

### Exposure to inhibitors of Ras signaling

Uninfected cells were exposed to Ras and its down-stream specific inhibitors overnight at the following concentrations: AG1478 at 0.5 µM, FTI-276 at 5 nM, PD98059 at 20 µM and Chromomycin A at 500 nM and then exposed to the virus. Chemicals were obtained from Calbiochem (CA).

### Non-radioactive affinity pull-down assay for ERK activation

Non-radioactive ERK pathway activation assay were performed in accordance to the manufacturer's (Cell Signaling, MA) instructions. Briefly, cells grown in 10 cm tissue culture dishes were lysed at 75–80% confluency with 300 ul of cell lysis buffer. Immobilized antibody-against phospho-ERK1/2 bead slurry capable of binding to the phosphorylated forms of ERK was then added to 200 µg of total cell protein in 200 ml of cell lysis buffer. The mixture was incubated with gentle rocking overnight at 4°C then collected, washed and introduced to an *in-vitro* kinase reaction in presence of ATP and ELK, the substrate for phospho-ERK. The levels of phosphorylated ELK was then assayed by western blotting using antibodies directed against phosphorylated (active) form of this transcription factor.

### Plaque (viral progeny) titration assay

Plaque titration was performed with the purpose of evaluating the yield of progeny virus at 24–48 hours post-infection. Briefly, the supernatant from infected cells were serially diluted from 1/10 to 1/10^8^. A volume of 300 µl from each dilution was added to duplicate wells (of 6-well plates) containing Vero cells at ∼75% confluency after removal of the existing media and rinsing the cells with phosphate the buffered saline (PBS). Cells were then incubated at 37°C until development of cytopathic effects in the form of plaques which usually occurs within 2–3 days. At this point, a monolayer was fixed with methanol and stained with Giemsa solution for 10 minutes. The number of clear plaques was then determined by calculating the average of number of plaques/well for each dilution and the volume used to infect each well.

### Immunofluorescent analysis of Herpes infection and analysis of antibody staining

Cells were grown in 8-well slide chambers (Falcon) and infected with HSV-1 (strain F) or SS1 or mock-infected. At different times post-infection, cells were fixed in acetone (100%) for 10 min and then left at room temperature to dry before incubation with a fluorescin-labeled mouse monoclonal antibody against HSV-1 gC antigen (labvision) for 30 min at 37°C. The slides were washed with distilled water, dried, and mounted in 90% glycerol containing 0.1% phenylenediamine, and viewed with a Zeiss Axiophot microscope on which a Carl Zeiss camera was mounted. Pictures were captured by an attached computer and processed with appropriate software. All images for phospho-ELK expression were analyzed and quantified by Image J. software (by National Institute of Health, NIH) which reflects brown nuclei staining due to the expression of phospho-ELK.

### Cell Invasion assay

In order to evaluate cell invasiveness, a commercial kit was used (BD Biosciences, CA). Briefly, cells (50,000 control or test cells) were introduced into Matrigel-coated inserts fitting 24 well plates. As cells invaded through the layer of Matrigel, the fraction of invaded cells were detected by staining them with crystal violet and quantifying them by spectroscopy. Invaded cells were fixed with 5% paraformaldehyde and stained with a 5%-solution of crystal violet and then photographed to obtain a visual representation of their density. The cells were then solublized in a 3%-detergent (NP40) solution, and the absorbance was measured by spectrophotometry at 590 nm.

### Cell proliferation assay

The cell proliferation assay was performed using a kit (Millipore, CA) according to the manufacturer's instructions. The assay is based on the cleavage of the tetrazolium salt WST-1 to formazan by cellular mitochondrial dehydrogenases. Expansion in the number of viable cells results in an increase in the overall activity of the mitochondrial dehydrogenases resulting in an increase in the amount of formazan dye formed. Briefly, 10^4^ cells/well were seeded in a 96-well microplate in volume of 100 µL/well. At different times, 10 µL WST-1/ECS solution was added to each well. The plates were incubated for 4 hours in standard culture conditions. The plates were then shaken thoroughly for one minute and absorbance was measured at 480 nm.

### Necrosis and apoptosis assay

Cells were seeded in a 96-well plate (plate-1) at a density of 1×10^4^ cells/well. After 24 hours cells were infected with SS-1 at MOI∼0.5 for 48 hr. The Plate-1 was centrifuged at 200 g for 10 min. Supernatant was introduced into another 96-well plate (plate-2) and kept it in 4°C. To lyse the cells in plate-1, 200 µl lysis buffer/well (Roche, Cat. No. 11774425001) was added. After incubation for 30 min at room temperature (RT), then plate-1 was centrifuged at 200 g for 10 min. 20 µl of lysates from plate-1 and 20 µl of supernatant from plate-2 each were introduced into the streptavidin coated microplates in triplicate for each sample. After 80 µl of the immunoreagent was added into each well, the microplate was covered and incubated on a shaker at 250 rpm for 2 hours at RT. In next step each well was rinsed 3 times with 250 µl incubation buffer and 100 µl ABTS solution was added and shaked 20 min at 250 rpm for color development. 100 µl ANTS stop solution was then added to each well and the emission was measured at 405 nm.

### Cell cycle assay

10^6^ cells for each cell line were pelleted and washed twice by CycleTEST PLUS buffer solution (BECTON DICKINSON, Cat. No. 340242). The cell pellet was then resuspended in 1 ml of the same buffer solution. To stain the cells, 250 µl Solution A and 200 µl solution B were added and incubated 10 minutes at RT. 200 µl of cold solution C was added in the last and incubated 10 minutes at 4°C. The cells were filtered through a 35-µm cell strainer cap into a 12×75-mm tube. The samples were kept the tube in dark and analyzed by FACSort flow cytometer.

### Colony formation assay

For colony formation assays, cells were infected with virus overnight with MOI∼1. Following infection, cells were trypsinized, counted and plated in triplicates at different concentration 100,200 –1000cells per well for each cell line in a 6 well plate and allowed to grow for 10–14 days changing media every 2 days. After 10–14 days when cells grew into visible colonies, plates were washed twice with PBS and were stained with crystal violet with methanol overnight. Next day plates were washed with water few times till the background was clear and number of colonies in different microscopic fields were counted.

### Statistical Analysis

Results are reported as means±standard deviation (SD). Student's *t* test was used to analyze statistical differences between groups. Alpha (α. level was set at 0.05).
